# Correspondence

**DOI:** 10.1155/S1463924682000480

**Published:** 1982


Journal of Automatic Chemistry, Volume 4, Number 4 (October–December 1982), pages 193–194

### Correspondence

### Flow-injection analysis. An idea complete—but yet far from fully exploited

*Correspondence from E. H. Hansen and Jaromir Ruzicka*

For reasons only known to himself Dr Holy of Technicon (in *Journal of Automatic Chemistry*, Vol. 4, No. 3, p. 111) has chosen to resume a discussion which was initiated by his colleague, Dr Margoshes, five years ago [1]. Despite the exchange of comments then [2] and the extensive volume of flow-injection analysis (FIA) publications since, Dr Holy's main arguments still remain indistinguishable from those of Dr Margoshes and therefore already have been answered—not only by us but even more by all those analysts who have used FIA for a variety of tasks and published their results in the literature. As we repeatedly have argued, it is the *concept of controlled dispersion* which is the thought underlying the theory and development of FIA and *not* the individual means, like method of injection, application of a flow-through detector or use of non-segmented streams [3]. In fact, it is naïve to confuse the discovery of this or any other new method with the tools and means of its experimental realization. These individual tools and means have, for FIA, been around for a long time—otherwise how could we have used them? It is only within the last few years we have learned to manipulate the dispersion and exploit it for analytical purposes, and therefore it is not surprising that the number of commercial instruments available (and sold) is to date limited. Hence, it is peculiar (albeit flattering) that Dr Holy pulls out of his hat the number of 50 000 (segmented) continuous flow units sold over a period of 25 years.

It is evident from the vast number of FIA publications that FIA methods yield highly reproducible analytical read-outs within *c*. 20 s. Therefore it is not a *must* to inject the individual samples and/or standards several times; rather, it is left to the analyst to balance, for routine purposes, the desired precision versus the sampling frequency. The ultimate aim of any instrumentation of 'sample in—result out in 1–2 min', as advanced by Dr Holy, has therefore already been achieved in FIA.

The advantages offered by FIA as compared to segmented continuous flow have been repeated so often in the literature that it is almost trivial to reiterate them. Enough to say that all methods based on exploiting the concentration gradient by the controlled dispersion (i.e. methods where the read-out is taken not only on the peak maximum, but on one [or several] position[s] of the concentration gradient, corresponding to element[s] of the introduced sample zone of different sample to reagent ratio[s]) are only feasible in non-segmented streams. Examples of such applications are stopped-flow reaction rate measurements, titrations, electronic dilution and calibration, gradient scanning, and selectivity evaluation methods.

Clark's law of revolutionary ideas [4] states that: 'every revolutionary idea—in science, politics, art or whatever—evokes three stages of reaction. They may be summed up by the three phrases: (1) "It is impossible—don't waste my time"; (2) "It is possible, but it is not worth doing"; (3) "I said it was a good idea all along"'.

If, however, one cannot understand the idea and therefore appreciate its novelty, the natural reaction will be: 'It was done a long time ago'!

We leave it to the readers to decide into which category Dr Holy's comments belong.

### References

1. Margoshes, M., *Analytical Chemistry*, **49** (1977), 17.
2. Ruzicka, J., Hansen, E. H., Mosbaek, H. and Krug, F. J., *Analytical Chemistry*, **49** (1977), 1858.
3. Ruzicka, J. and Hansen, E. H., *Flow Injection Analysis* (John Wiley and Sons, New York, 1981).
4. Block, A., *Murphy's Law* (Price Publications Inc., Los Angeles, 1977).

*Chemistry Department A, The Technical University of Denmark, Building 207, DK-2800 Lyngby, Denmark*

### A reply to H. W. Holy

*Correspondence from Kent K. Stewart*

H. W. Holy's commentary entitled 'Flow-injection analysis—an idea incomplete?' (*Journal of Automatic Chemistry*, Vol. 4, No. 3, p. 111) is a curious mixture of tunnel vision and some nice insight into some basic questions in automation.

First, the literature base for the articles; in a comment on flow-injection analysis (FIA), it is curious that nothing more recent than 1959 is mentioned.* Much more exists. My reference file has over 200 references and there have been a number of recent reviews [1–5] and one textbook [6]. Curious that these are not mentioned.

In regard to FIA theory, I am in complete agreement that the early work of Taylor and Aris laid the foundation for a theoretical understanding of dispersion in a flowing stream. However, while there is not yet complete agreement about dispersion in FIA systems, there has been a considerable amount of work in this area [7–10]. There does seem to be complete agreement that in FIA systems the flow is completely laminar and *not* turbulent [5]. Thus, the claim by Dr Holy that the goal of FIA is achievable 'given completely turbulent flow' does *not* match the results of recent studies.

Dr Holy's discussion about duplicate assays seems unfortunate. All assays should be run in duplicate; not for statistical reasons, but to minimize the influence of random

*Letters in JAC express their authors' views only and it should not be considered that the Editor and his advisers necessarily agree with them.*

* *I am grateful to Dr Holy for bringing the article by Jonnard to my attention. It is a rather obscure paper; I was unable to find any citation to it in the 'Science Citation Indexes' from 1970 to 1982.*

disasters. The availability of a rapid, precise system with a high throughput, such as FIA, to ease the burden of duplicate assays should be a positive asset to any analyst.

The key question asked by Dr Holy was 'What has been gained?' Those of us who work with FIA believe that FIA offers an exciting means of rapidly doing a number of different assays with greater throughput, better precision and accuracy and less operator time. It is *not* a matter of 'analysing of metals with FIA rather than atomic absorption'; it is a matter of determining metal content with FIA *and* atomic absorption [11]. I see FIA as a young child with enormous potential, while CFA appears to be a mature field approaching middle age.

When I remember Dr Holy is an employee of Technicon I am inclined to say 'Me thinks the gentleman does protest too much'.

*Department of Food Science and Technology, College of Agriculture and Life Sciences, Virginia Polytechnic Institute and State University, Blacksburg, Virginia 24061, USA*

### References

1. Betteridge, D., *Analytical Chemistry*, **50** (1978), 832A.
2. Ruzicka, J. and Hansen, E. H., National Bureau of Standards Special Publication 519: *Trace Organic Analysis: A New Frontier in Analytical Chemistry. Proceedings of the 9th Materials Research Symposium* (held at NBS, Gaithersburg, Maryland, April 1978), 501.
3. Ruzicka, J. and Hansen, E. H., *Analytica Chimica Acta*, **114** (1980), 19.
4. Ranger, C. B., *Analytical Chemistry*, **53** (1981), 20A.
5. Stewart, K. K., *Talanta*, **28** (1981), 789.
6. Ruzicka, J. and Hansen, E. H., *Flow Injection Analysis* (John Wiley & Sons, New York, 1981).
7. Ruzicka, J. and Hansen, E. H., *Analytica Chimica Acta*, **99** (1978), 37.
8. Reijn, J. M., *Analytica Chimica Acta*, **114** (1980), 105.
9. Vanderslice, J. T., Stewart, K. K., Rosenfeld, A. G. and Higgs, D. J., *Talanta*, **28** (1982), 1.
10. Pardue, H. L. and Fields, B., *Analytica Chimica Acta*, **124** (1981), 39 and 65.
11. Wolf, W. R. and Stewart, K. K., *Analytical Chemistry*, **51** (1979), 1201.

### Data handling on a SMAC

*Correspondence from Charles R. Midkiff*

In the article by Dugdale, Harrison and Robertshaw, 'Data handling on a sequential multi-channel analyzer computerized (SMAC): using a low-cost minicomputer' in the January–March 1982 issue of the *Journal*, there is a minor misconception. The computer used, the Exidy Sorcerer, is not, as indicated, a minicomputer. It is more properly designated as a personal computer or microcomputer.

While it is true that, with the new generation of microcomputers, the distinctions between mini- and microcomputors are becoming blurred, in general the distinction is made based upon the central processing unit (CPU). As James Tuttle, director of development for Data General's small business systems division put it 'If you draw a distinction on the engines in the two, a microcomputer is a one-chip processor, while a mini can expand over memory sizes and peripherals'. Although minicomputers are being developed which use one-chip processors (engines), this is not currently state-of-the-art. In addition, most minicomputers use 16 bit or greater CPUs, whereas 16-bit microcomputers are a recent development and at present represent only a very limited portion of the total market for personal computers.

Both the Exidy Sorcerer used by the authors and the Commodore PET, mentioned by them, are based upon 8-bit microprocessors. The CPU in the Exidy Sorcerer is a Z-80 made by Zilog Inc. and that of the PET is a 6502 made by Commodore. Each is represented and sold by the manufacturer as a microcomputer.

Because of the great contributions which can be made to the laboratory by computers, particularly the low-cost microcomputer, I feel that it is important that, at this stage in the development of automated chemical methods, it be made clear to the reader the type of system used.

Overall, I feel that the authors illustrated well one role that low-cost computer systems can play in the laboratory. Because they are so versatile, these computers can readily handle data collection, filing, calculation and report generation. They will be an integral part of every laboratory in the near future.

*Chemical Branch, Department of the Treasury, Bureau of Alcohol, Tobacco and Firearms, 1401 Research Boulevard, Rockville, Maryland 20850, USA*

### SEMINAR ANNOUNCEMENT

*'Optical sensing: techniques, benefits and costs' (7–8 December 1982), to be held at NPL, Teddington, Middlesex, UK*

Organized jointly by the National Physical Laboratory (NPL) and Sira, this seminar will be of particular interest to technical directors; to research, development and design engineers; and to production and quality-control managers. It will emphasize cost-effective applications of optical techniques in the fields of metrology, inspection and process control, including new types of optical sensor and developments in electronic vision. The technical sessions will cover: engineering metrology (metrology and inspection, machine monitoring, process gauging), and process control. Each technical session will be introduced by an invited review paper, followed by several shorter contributions. The seminar is sponsored by the Institute of Measurement and Control and the Institution of Electrical Engineers.

*For further information about the seminar and the accompanying exhibition of optical measuring equipment contact the Commercial Department, Sira Institute Ltd, South Hill, Chislehurst, Kent BR7 5EH, UK. Tel.: 01 467 2636.*

